# Hepatic Rather Than Cardiac Steatosis Relates to Glucose Intolerance in Women with Prior Gestational Diabetes

**DOI:** 10.1371/journal.pone.0091607

**Published:** 2014-03-12

**Authors:** Yvonne Winhofer, Martin Krššák, Peter Wolf, Andrea Tura, Christian-Heinz Anderwald, Lana Kosi, Gert Reiter, Giovanni Pacini, Siegfried Trattnig, Anton Luger, Michael Krebs, Alexandra Kautzky-Willer

**Affiliations:** 1 Department of Internal Medicine III, Division of Endocrinology and Metabolism, Medical University of Vienna, Vienna, Austria; 2 Metabolic Unit, Institute of Biomedical Engineering, National Research Council, Padova, Italy; 3 Mariahilf Community Pharmacy, Arnoldstein, Austria; 4 Medical Direction, Specialized Hospital Complex Agathenhof, Micheldorf, Austria; 5 Siemens AG Healthcare, Vienna, Austria; 6 Centre of Excellence, High-Field MR, Department of Radiodiagnostics, Medical University of Vienna, Vienna, Austria; University of Basque Country, Spain

## Abstract

**Background:**

Increased myocardial lipid accumulation has been described in patients with pre- and overt type 2 diabetes and could underlie the development of left-ventricular dysfunction in metabolic diseases (diabetic cardiomyopathy). Since women with prior gestational diabetes (pGDM) display a generally young population at high risk of developing diabetes and associated cardiovascular complications, we aimed to assess whether myocardial lipid accumulation can be detected at early stages of glucose intolerance and relates to markers of hepatic steatosis (Fatty Liver Index), cardiac function, insulin sensitivity and secretion.

**Methods:**

Myocardial lipid content (MYCL), left-ventricular function (^1^H-magnetic-resonance-spectroscopy and -imaging), insulin sensitivity/secretion (oral glucose tolerance test) and the fatty liver index (FLI) were assessed in 35 pGDM (45.6±7.0 years, 28.3±4.8 kg/m^2^) and 14 healthy control females (CON; 44.7±9.8 years, 26.1±2.5 kg/m^2^), matching for age and body-mass-index (each p>0.1).

**Results:**

Of 35 pGDM, 9 displayed normal glucose tolerance (NGT), 6 impaired glucose regulation (IGR) and 20 had been already diagnosed with type 2 diabetes (T2DM). MYCL and cardiac function were comparable between pGDM and CON; in addition, no evidence of left-ventricular dysfunction was observed. MYCL was inversely correlated with the ejection fraction in T2DM (R = −0.45, p<0.05), while the FLI was tightly correlated with metabolic parameters (such as HbA1C, fasting plasma glucose and HDL-cholesterol) and rose along GT-groups.

**Conclusions:**

There is no evidence of cardiac steatosis in middle-aged women with prior gestational diabetes, suggesting that cardiac complications might develop later in the time-course of diabetes and may be accelerated by the co-existence of further risk factors, whereas hepatic steatosis remains a valid biomarker for metabolic diseases even in this rather young female cohort.

## Introduction

Increased myocardial lipid content (cardiac steatosis) is regarded as an important feature of disturbed myocardial substrate metabolism and thought to underlie the development of heart failure especially in patients with metabolic diseases, like diabetes (diabetic cardiomyopathy) [1], [2]. In analogy to ectopic lipid deposition in liver and skeletal muscle, myocardial lipid content (MYCL) can nowadays be non-invasively assessed by ^1^H magnetic resonance (MR) spectroscopy [3], [4]. MR imaging furthermore displays the method of choice for determining systolic cardiac function [5].

Prior investigations applying cardiac MR-spectroscopy revealed myocardial lipid content to be increased in patients with type 2 diabetes and some authors also reported cardiac steatosis in pre-diabetic subjects [6]. In addition, excessive lipid accumulation within the myocardium was found to be associated with elevated left ventricular mass [7] and diastolic dysfunction [2], both key features of the diabetic cardiomyopathy. Interestingly, it has been recently reported that - following the onset of overt diabetes - women exhibit more pronounced cardiac steatosis compared to men with type 2 diabetes [8].

We recently showed that cardiac steatosis is unrelated to insulin resistance in healthy women with normal glucose tolerance [9]. On the other hand, we demonstrated that combined hyperglycemia and hyperinsulinemia increase myocardial lipid content in healthy subjects, suggesting that this environment typical for pre- and early type 2 diabetes might be responsible for the development of cardiac steatosis [10].

Women with prior gestational diabetes (pGDM) display a relatively young population at high risk of developing diabetes. Furthermore, glucose intolerance during pregnancy is associated with an increased risk for cardiovascular disease including premature cardiovascular events [11]–[14]. Since pGDM exhibit many features of the *Cardio-metabolic Syndrome*, including hyperglycemia and hyperinsulinemia [15], [16] we hypothesized that cardiac steatosis might present an early sign of cardiac vulnerability and can be detected in these women with impaired glucose tolerance and early diabetes.

Therefore, the aim of this study was to investigate myocardial lipid content and cardiac function and its relations to other features of the *Cardio-metabolic Syndrome*, such as fatty liver, insulin insensitivity and altered insulin secretion in women with prior gestational diabetes, when compared to healthy controls.

## Materials and Methods

### Study population

Thirty-five women with prior gestational diabetes (pGDM) were recruited from the 10-years-follow-up visit of the „Viennese Post-Gestational Diabetes Project (VPGDP)” [17] a prospective observational study, and from the diabetes outpatient clinic, to be compared to 14 women without a history of glucose intolerance (healthy controls, CON). All pGDM had a history of GDM and no evidence for autoimmune diabetes, confirmed by the assessment of diabetes-specific antibodies during pregnancy and postpartum. Exclusion criteria were several cardiac diseases including coronary artery disease, valvulopathy, congenital heart disease and a history of myocarditis; furthermore, severe arterial hypertension, the intake of thiazolidiones and morbid obesity (defined by a BMI ≥40 kg/m^2^ or a BMI ≥35 kg/m^2^ with obesity-related health conditions such as diabetes or arterial hypertension).

pGDM were divided into three groups according to their medical history (known type 2 diabetes, antihyperglycemic therapy) and their glucose tolerance status during a 75 g-oral glucose tolerance test (OGTT) based on the criteria of the American Diabetes Association (ADA): Thereby overt diabetes is diagnosed by fasting plasma glucose ≥126 mg/dl and/or 2-h-plasma glucose ≥200 mg/dl, impaired glucose tolerance (IGT) is defined by a 2-h-plasma glucose between 140 and 199 mg/dl and impaired fasting glucose (IFG) by a fasting plasma glucose between 100 and 125 mg/dl. IGT and IGF were summarized under the term “impaired glucose regulation (IGR)”. In pGDM with known type 2 diabetes, no OGTT was performed. From the 35 pGDM, 9 were diagnosed with normal glucose tolerance (NGT), 6 with IGR and 20 had already developed type 2 diabetes (T2DM).

The human ethics committee of the Medical University of Vienna approved the protocol and all women gave written informed consent.

### Assessment of myocardial lipid content

ECG-gated ^1^H-NuclearMR-spectroscopy was used to assess myocardial lipid content similarly to previously described protocols [2], [4] by positioning the volume of interest (VOI; approx. 6–8 cm^3^) in the interventricular septum to avoid signal alterations by the epicardial fat. Water suppressed Point RESolved Spectroscopy (PRESS) sequence (echo time, TE = 30 ms, number of averages [NS] = 8–12, within one breath hold, repetition time TR = 700–1250 ms according to heart rate) was used for spatial localization and signal acquisition. An additional spectrum without water suppression (NS = 4) was used as the internal concentration reference. The spectra were processed via the spectroscopy processing tool provided by the system manufacturer (Siemens, Erlangen, Germany) and the myocardial lipid content is expressed as ratio of the sum intensities of methylene (CH_2_; 1.3 ppm) and methyl (CH_3_, 0.9 ppm) resonance lines to that of water signal. T1 and T2 relaxation correction was performed using the T1 and T2 values measured at 3T in skeletal muscle.

### Assessment of left ventricular function by MR-imaging

Visualization of cardiac function was performed using retrospectively ECG-gated cine true fast imaging with steady state free precession (True FISP) sequences in 2-, 4-chamber and short axes orientation, in the latter orientation with 10–12 equidistant imaging levels from the apex to the base of the left ventricle. Ejection fraction, end-systolic and end-diastolic volume, cardiac output, stroke volume, left ventricular mass (LVM) [4] as well as concentricity ( = LVM to end-diastolic volume ratio (LVM/LVEDV) [18]) were calculated by dedicated manufacturer's software (ARGUS, Siemens, Erlangen, Germany) after manually contouring endo- and epicardial borders in end-systolic and end-diastolic short axes cine images of the left ventricle. Papillary muscles were counted to muscle mass and data are presented normalized to body surface area (BSA) using the Dubois formula (BSA = 0.007184×height^0.725^×weight^0.425^). After exclusion of papillary muscles contours were additionally used for assessment of left ventricular wall thickness [19] according to the 16-segment model of the ASE. The presented means of left-ventricular wall thickness and myocardial mass were calculated at end-diastole.

Furthermore a retrospectively ECG-gated, spoiled gradient echo–based phase-contrast sequence (through-plane velocity encoding typically 90 cm/s) was employed to assess blood flow across the mitral valve. Biphasic diastolic inflow patterns consisting of 2 peaks, representing the peaks of the early filling phase (E) and the atrial contraction (A), were evaluated by dedicated manufacturer's software (ARGUS, Siemens, Erlangen, Germany) and are here presented as: E/A-ratio, a marker of diastolic function [20].

All MR and metabolic examinations were performed after an overnight fast for at least 8 hours. MR-examinations took place on a 3.0-T scanner (Magnetom Trio, Siemens, Erlangen, Germany), employing a (standard) 6-channel body matrix coil.

### Oral glucose tolerance test (OGTT)

Concentrations of glucose, insulin and C-peptide were measured at fasting as well as 30, 60, 90, 120, 150 and 180 minutes after ingestion of a 75 g-glucose-solution. Blood samples were drawn over a catheter placed in one ante-cubital vein.

The kinetics of glucose, insulin and C-peptide during OGTT were analyzed by quantitative methods to obtain metabolic parameters, such as insulin sensitivity through OGIS, that describes glucose clearance per unit change of insulin concentration [21]. Areas under the curve (AUC) of C-peptide (AUC_Cpep) and insulin (AUC_Ins) during the whole test, calculated with the trapezoidal rule, represent indices of insulin secretion. Percent hepatic extraction (HIE), calculated for the whole period of the test (and not only during the first pass) was determined according to the model described previously [22]. Briefly, it is given by first subtracting from the beta cell release (k01×AUC_Cpep) the post-hepatic appearance (n×AUC_Ins) and then dividing this difference by 10^−2^×k01×AUC_Cpep. Parameters k01 and n are the fractional clearances (min^−1^) of C-peptide and insulin, respectively; they have been determined previously from arterio-venous differences across the liver and vary according to the metabolic status of the subject.

At fasting also blood pressure (in mmHg) was measured and blood samples for the assessment of hemoglobin A1C (HbA1C), triglycerides, total cholesterol, HDL-cholesterol, LDL-cholesterol and high sensitive CRP (hsCRP) were drawn.

Insulin and C-peptide were measured by IMMULITE 2000 immunoassay system with a CV within-run <5,5% and total <7,3% for insulin and a CV within-run <2,3% und total <4,8% for C-peptide. High-sensitive CRP (N High Sensitivity CRP Reagent, BN™ Systems, Dade Behring, Deerfield, IL), glucose, HbA1C, triglycerides, total cholesterol, HDL- and LDL-cholesterol were assessed by established methods in the central lab of the Medical University of Vienna.

### Fatty Liver Index (FLI)

The FLI was calculated by the previously validated formula [23]:

FLI  =  (e ^0.953*loge (triglycerides) +0.139*BMI +0.718*loge (ggt) +0.053*waist circumference −15.745^) / (1+ e ^0.953*loge (triglycerides) +0.139*BMI +0.718*loge (ggt) +0.053*waist circumference −15.745^) * 100

with body mass index (BMI) in kg/m^2^, gamma-glutamyltransferase (ggt) in U/l, waist circumference in cm and triglycerides in mg/dl.

### Accuracy of the FLI

In order to evaluate the accuracy of the fatty liver index in our study group, the assessment of hepatic lipid content (HCL) by MR-spectroscopy was optional and offered to all study participants. HCL was measured in analogy to previously published protocols [24], [25]. Since the majority of control subjects did not undergo the optional MR-examination, data were analyzed from pGDM with normal glucose tolerance (NGT, n = 9) compared to those pGDM with type 2 diabetes (T2DM, n = 20).

### Medication records

Of the 20 pGDM with type 2 diabetes, 12 were on insulin therapy and 8 T2DM on oral anti-glycemic agents only, which included metformin, sulfonylurea and dipeptidyl peptidase 4-inhibitors. In addition, antihypertensive- and lipid lowering (statins, ezetimibe) medication was recorded. No woman in this study was taking thiazolidiones.

### Statistical analysis

Comparisons of quantitative variables among groups were performed using ANOVA with Tukey's studentized range test (HSD) for post-hoc analyses. Pearson's correlation analysis was used to describe associations between continuous variables. Levels of statistical significance were set at p<0.05. All tests were performed using SAS® Enterprise guide® 4.3.

## Results

### Baseline characteristics

Baseline characteristics including age, BMI and blood pressure were comparable between the groups, while indices of hypergylcemia were significantly increased in the T2DM-group (see **Table 1**).

**Table 1:** Baseline characteristics of the whole study group: healthy controls (CON) and women with prior gestational diabetes (pGDM) with normal glucose tolerance (NGT), impaired glucose regulation (IGR) and overt type 2 diabetes (T2DM); ns = non-significant; significant differences according to ANOVA with Tukey's as post-hoc test are marked with * for differences between T2DM and all other groups, ** for differences between T2DM and NGT as well as CON, † for differences between IGR compared to NGT and CON and ‡.for differences between T2DM and CON.

	CON	NGT	IGR	T2DM	p-value
n	14	9	6	20	
Age(years)	44.7±9.8	44.0±4.0	41.9±4.0	47.4±8.3	ns
Body-Mass-Index (kg/m^2^)	26.1±2.5	27.3±5.4	28.9±5.7	28.5±4.5	ns
Body-Surface-Area (m^2^)	1.8±0.1	1.8±0.1	1.8±0.2	1.8±0.2	ns
Systolic blood pressure (mmHg)	119±15	111±9	123±22	126±15	ns
Diastolic blood pressure (mmHg)	79±10	74±9	81±13	79±8	ns
HbA1C(%)	5.5±0.3	5.4±0.3	5.4±.3	8.0±1.6	<.0001*
Fasting glucose (mg/dl)	89.3±7.5	86.9±10.0	102.0±18.2	136.5±56.2	<0.002**
2-h postprandial glucose (mg/dl; OGTT)	95.4±24.3	110.3±11.7	168.7±17.5	nd	p<.0001†
Triglycerides (mg/dl)	91.7±20.3	107.1±40.9	104.7±28.7	232.6±220.9	0.04‡
Total cholesterol (mg/dl)	224.7±29.2	202.5±37.7	200.0±23.1	205.2±63.7	ns
LDL-cholesterol (mg/dl)	134.3±27.3	114.7±30.6	123.2±20.8	123.6±43.5	ns
HDL-cholesterol (mg/dl)	72.1±16.9	66.4±17.6	55.8±12.7	48.3±12.3	0.0006**
Fatty Liver Index	30.1±18.6	42.0±30.5	53.3±32.9	62.0±30.9	0.03‡

### Comparisons between groups

**Figure 1:**
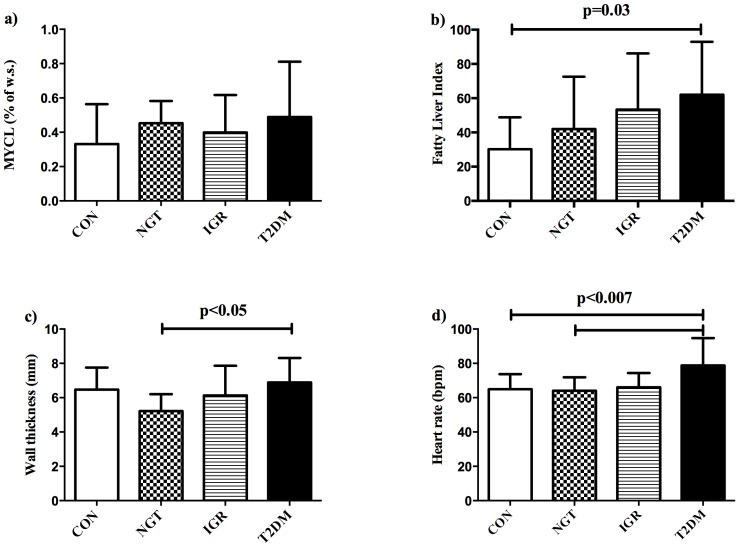
Comparison of metabolic and cardiac parameters between the groups. **a**) Myocardial lipid content (MYCL) was comparable between the groups: healthy controls (**CON**), pGDM with normal glucose tolerance (**NGT**), pGDM with impaired glucose regulation (**IGR**) and pGDM with overt type 2 diabetes (**T2DM**), while **b**) the Fatty Liver Index (FLI) increased along the GT-groups and was significantly increased in T2DM compared to CON; **c**) left-ventricular wall thickness was higher in T2DM compared to NGT and **d**) heart rate was highest in T2DM, being significantly different from NGT and CON.

Myocardial lipid content was comparable between the groups (see **Fig.1a**). The Fatty Liver Index (FLI) gradually increased along the groups (CON < NGT < IGR < T2DM), and was significantly different between CON and T2DM (p = 0.03, see **Table 1** **and** **Fig.1b**). In pGDM who additionally underwent MR-spectroscopy of the liver, hepatic lipid content (HCL) was tightly correlated with FLI: R = 0.67, p = 0.0002 and 2-times higher in T2DM compared to NGT (5.6±5.8 vs. 2.4±2.4% of w.s., p = ns).

There was no difference in metabolic or spectroscopy data (including FLI, HCL and MYCL) between T2DM treated with insulin and those treated with oral agents.

Left ventricular wall thickness was higher in T2DM compared to NGT (see **Fig. 1c**), but did not differ when compared to IGR and CON. Heart rate (**Fig.1d**) was higher in T2DM than NGT and CON, but not IGR. The E/A-ratio, as marker of diastolic function, and the concentricity-index, which describes myocardial remodeling, as well as markers of left-ventricular systolic function (ejection fraction, stroke volume, cardiac index) were comparable among the groups.

### Correlation analyses

**Figure 2:**
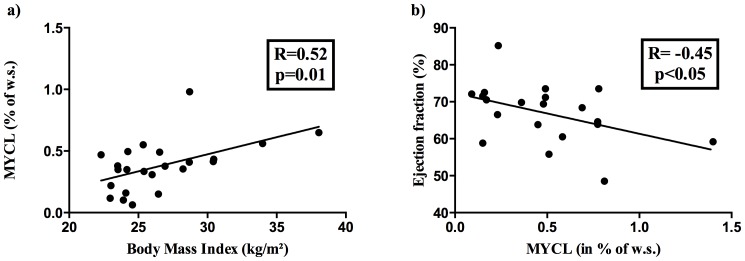
Correlation analysis. **a**) Myocardial lipid content (MYCL) was positively correlated with the body mass index in CON and NGT and **b**) inversely associated with the ejection fraction in T2DM.

There was no association between MYCL and metabolic parameters, including insulin sensitivity (assessed by OGIS), in the whole study group, while grouped analyses showed that MYCL was significantly correlated with the body mass index in CON and NGT (see **Fig.2a**) and in NGT, MYCL was additionally associated with diastolic blood pressure (R = 0.81, p<0.008) and left-ventricular mass (R = 0.77, p = 0.01). In T2DM, MYCL was inversely correlated with the ejection fraction (see **Fig. 2b**).

The FLI was positively correlated with parameters of hyperglycemia including fasting plasma glucose (R = 0.49, p = 0.0009), 2-h-postprandial glucose (R = 0.46, p = 0.01) and HbA1C (R = 0.48, p = 0.001) and, furthermore, inversely associated with HDL-cholesterol (R = -0.73, p<.0001) in the whole study group. In those who underwent an OGTT, FLI was additionally associated with fasting concentrations of C-peptide (R = 0.42, p = 0.03) and inversely with the hepatic insulin extraction (R = −0.44, p = 0.02).

Myocardial mass was positively correlated with systolic blood pressure (R = 0.48, p = 0.0006), while left-ventricular wall thickness was correlated with systolic (R = 0.54, p = 0.0002) and diastolic (R = 0.50, p = 0.0007) blood pressure, BMI (R = 0.45, p = 0.002), HbA1C (R = 0.40, p = 0.008), the AUC of C-peptide during the OGTT (R = 0.54, p<0.009), high sensitive CRP (R = 0,7, p<0.002) and inversely with HDL-cholesterol (R = −0.39, p<0.02).

## Discussion

The current study aimed to assess whether women with prior gestational diabetes (pGDM) - at different stages of glucose intolerance - already exhibit features of incident cardiac steatosis, predisposing them for the development of (diabetic) cardiomyopathy. According to our data, neither myocardial lipid content nor left ventricular function differed between pGDM and healthy controls. In addition, none of the groups showed evidence of cardiac steatosis or cardiac dysfunction, indicating that metabolic disturbances might not influence cardiac morbidity in this relatively young female population. In contrast to prior investigations in patients with diabetes [2], we could not detect a link between MYCL and diastolic function, assessed by the E/A-ratio. Our results are also in contrast to a prior investigation, which reported increased MYCL in women with diabetes [8]. However, in both studies male patients were over-represented and, moreover, the study populations were about 10–15 years older than ours. Assuming that the development of cardiomyopathy in patients with diabetes may take years and furthermore be accelerated by the co-existence of arterial hypertension and coronary artery disease [26], our study population might be too young to detect cardiac abnormalities.

In addition, myocardial lipid content was independent of medication intake (insulin versus oral anti-glycemic agents) and not associated with insulin sensitivity, described by OGIS. This is in line with our prior studies, in which we also could not find a link between insulin resistance and myocardial lipid accumulation in healthy women [9]. Furthermore, we have previously shown that combined hyperglycemia and hyperinsulinemia increase myocardial lipid content in both, healthy men and women, and that myocardial lipid accumulation tightly relates to hyperinsulinemia. However, MYCL was not associated with insulin sensitivity, calculated by the M-value during the clamp, and its increase comparable between male and female subjects [10].

In contrast to our findings, another group has described a link between insulin resistance and cardiac steatosis in sedentary, obese women; subsequently we assume that this might be related to increased adiposity in those women with a mean BMI of 33 kg/m^2^ [27]. This assumption is supported by our data, which showed a positive correlation between BMI and MYCL in the CON- and NGT-group, both with normal glucose tolerance. Thus, the co-existence of obesity might accelerate myocardial lipid accumulation due to increased endogenous fatty acids and insulin supply, at least in women without disturbed glucose metabolism.

Correlation analyses revealed a weak, but significant inverse association between MYCL and systolic left-ventricular function in the T2DM-group. However, the ejection fraction did not differ between the groups. The link between myocardial lipids and systolic function is of interest, since myocardial lipid accumulation has been linked to the development of diabetic cardiomyopathy triggered by lipotoxicity [28], [29]. Until now, our knowledge mainly relies on animal data, and recent reports have focused on the quality rather than the quantity of myocardial lipids; there is evidence that in particular saturated free fatty acids (SFA) might favor the development of cardiac hypertrophy and dysfunction [30], while unsaturated free fatty acids might play a protective role. Hence, it can be speculated that the assessment of lipid composition rather than quantification of triglycerides in the heart alone, might help to draw a link between myocardial lipid accumulation and cardiac dysfunction.

In our current study, we found that besides blood pressure, myocardial wall thickness was tightly associated with BMI, glycemic control (HbA1C), hyperinsulinemia (AUC of C-peptide during the OGTT) and hsCRP. Again, we think that long-term exposure to these co-existing risk factors might accelerate the development of myocardial hypertrophy in patients with diabetes.

In contrast to myocardial lipids, hepatic steatosis, estimated by the Fatty Liver Index, was tightly correlated with metabolic parameters: glycemic control (HbA1C and fasting plasma glucose), hyperinsulinemia (fasting C-peptide) as well as dyslipidemia (low HDL-cholesterol). In a recent study in women with prior gestational diabetes [31], hepatic lipid content (assessed by MR-spectroscopy) was doubled in pGDM compared to healthy controls and tightly associated with insulin resistance. Thus, we conclude that hepatic lipid accumulation better reflects gluco-metabolic alterations than do myocardial lipids. This could be explained by a higher turnover of the myocardial lipid pool compared to that of the liver.

There are some study limitations that have to be addressed. First of all, the study group is rather small in terms of participants, due to the difficulty of recruitment from the long-lasting project with a well-characterized cohort and the cost- and time-consuming examinations. Secondly, we investigated a quite young, pre-menopausal female population, whose cardiovascular risk factors might still be low to detect relevant changes. Furthermore, diastolic function is only described by the E/A-ratio, which might limit our conclusions on diastolic function.

### Conclusions

This study clearly shows that there is no evidence of cardiac steatosis or left-ventricular dysfunction in middle-aged women with prior gestational diabetes, indicating, that cardiac mal-adaptation to metabolic disarranges remains obscure for years before the onset of clinically relevant cardiac dysfunction. In addition, the co-existence of obesity, arterial hypertension and coronary artery disease might accelerate the onset of cardiac complications (steatosis, ventricular dysfunction) in patients with diabetes. Our data seem to confirm, that myocardial lipid content is not related to insulin sensitivity and showed that it is not an early derangement in women with pGDM. Thus, diabetes prevention programs may help to delay or prevent cardiac steatosis in pGDM. On the other hand, markers of hepatic lipid accumulation can be detected even in pGDM with normal glucose tolerance and closely relate to disturbances of glucose and lipid metabolism in such a relatively young female cohort at increased risk of cardio-metabolic diseases.
